# seGMM: A New Tool for Gender Determination From Massively Parallel Sequencing Data

**DOI:** 10.3389/fgene.2022.850804

**Published:** 2022-03-03

**Authors:** Sihan Liu, Yuanyuan Zeng, Chao Wang, Qian Zhang, Meilin Chen, Xiaolu Wang, Lanchen Wang, Yu Lu, Hui Guo, Fengxiao Bu

**Affiliations:** ^1^ Institute of Rare Diseases, West China Hospital of Sichuan University, Chengdu, China; ^2^ School of Medicine, National Institute for Data Science in Health and Medicine, Xiamen University, Xiamen, China; ^3^ Center for Medical Genetics and Hunan Provincial Key Laboratory of Medical Genetics, School of Life Sciences, Central South University, Changsha, China

**Keywords:** massively parallel sequencing data, Gaussian mixture model, gender, sex chromosomal abnormality, aneuploidy

## Abstract

In clinical genetic testing, checking the concordance between self-reported gender and genotype-inferred gender from genomic data is a significant quality control measure because mismatched gender due to sex chromosomal abnormalities or misregistration of clinical information can significantly affect molecular diagnosis and treatment decisions. Targeted gene sequencing (TGS) is widely recommended as a first-tier diagnostic step in clinical genetic testing. However, the existing gender-inference tools are optimized for whole genome and whole exome data and are not adequate and accurate for analyzing TGS data. In this study, we validated a new gender-inference tool, seGMM, which uses unsupervised clustering (Gaussian mixture model) to determine the gender of a sample. The seGMM tool can also identify sex chromosomal abnormalities in samples by aligning the sequencing reads from the genotype data. The seGMM tool consistently demonstrated >99% gender-inference accuracy in a publicly available 1,000-gene panel dataset from the 1,000 Genomes project, an in-house 785 hearing loss gene panel dataset of 16,387 samples, and a 187 autism risk gene panel dataset from the Autism Clinical and Genetic Resources in China (ACGC) database. The performance and accuracy of seGMM was significantly higher for the targeted gene sequencing (TGS), whole exome sequencing (WES), and whole genome sequencing (WGS) datasets compared to the other existing gender-inference tools such as PLINK, seXY, and XYalign. The results of seGMM were confirmed by the short tandem repeat analysis of the sex chromosome marker gene, amelogenin. Furthermore, our data showed that seGMM accurately identified sex chromosomal abnormalities in the samples. In conclusion, the seGMM tool shows great potential in clinical genetics by determining the sex chromosomal karyotypes of samples from massively parallel sequencing data with high accuracy.

## Introduction

The next-generation sequencing (NGS) technology has revolutionized human biology and medicine in the last decade. NGS is routinely used in clinical genetic testing for molecular diagnosis of hereditary disorders, infectious diseases, and immune disorders, non-invasive prenatal genetic testing, and personalized precision medicine, especially for cancer patients (Phillips and Douglas, 2018; Phillips et al., 2020). Clinical genetic testing is a diagnostic tool that involves genome sequencing to identify pathogenic gene mutations (genetic variants) in human diseases (McPherson, 2006). This may involve targeted gene sequencing (TGS) of single or multiple genes, whole exome sequencing (WES), or whole genome sequencing (WGS) (Di Resta et al., 2018). TGS is highly accurate, robust, and cost-effective. Therefore, TGS has been used for the diagnosis of several human diseases including hearing loss, vision loss, cardiovascular disorders, neurologic disorders, cancer risk, and renal disorders (Lin et al., 2012; Saudi Mendeliome, 2015).

Parallelized TGS analysis of large patient cohorts requires rigorous quality control (QC) and preprocessing to identify the pathogenic gene variants (Lee et al., 2017). Verification of the concordance between self-reported gender and genetically inferred gender is an essential QC step because misregistration of clinical information, sample swaps, sample pollution, or sex chromosomal abnormalities can result in wrong conclusions and affect treatment decisions (Taylor et al., 2015; Webster et al., 2019). Sex chromosomal abnormalities are reported in approximately 1 in 448 newborn children (Nielsen and Wohlert, 1990). Therefore, there is a higher probability of gender inconsistencies in larger cohorts. Cytogenetic karyotyping is the gold standard method for confirming the gender of an individual and identifying chromosomal abnormalities. The highly conserved sex chromosomal marker gene, amelogenin, is widely used for identifying gender using short tandem repeat (STR) typing (Thangaraj et al., 2002; Ma et al., 2012). The 6 bp deletion within intron 1 of the amelogenin gene in the X chromosome is used to distinguish the PCR amplified products of the amelogenin gene in the X and Y chromosomes (Sullivan et al., 1993). However, these methods are time- and labor-consuming.

Several computational tools such as PLINK, seXY, and XYalign, have been developed for gender inference based on genome-wide WES or WGS data. PLINK inferred gender by calculating F coefficients from the genotyping array data using X chromosome homozygosity/heterozygosity rates; samples with F coefficient values of more than 0.8 were designated as males and samples with F coefficient values of less than 0.2 were considered as females (Purcell et al., 2007). The seXY tool is based on the logistic regression model and identifies gender by considering X chromosome heterozygosity and Y chromosome missingness in the genotyping array data (Qian et al., 2017). XYalign tool identifies gender from both WES and WGS datasets by extracting the read counts mapped to the sex chromosomes and calculating the ratio of X and Y read counts in a scatter plot (Webster et al., 2019). However, none of these tools are optimized for analyzing TGS panel data, which contains significantly reduced information compared to the whole genomic or exome data. As shown in Table 1, the performance of the existing tools was not satisfactory in reporting gender using the TGS data. Furthermore, sex chromosomal abnormalities were not clearly identified by the PLINK, seXY, and XYalign tools. Few studies reported the sex chromosomal abnormalities of individuals based on the ratio of sequencing reads that were mapped to the X and Y chromosomes from the genotyping array and WGS data (Bycroft et al., 2018; Turro et al., 2020). However, this methodology has not been automated. Therefore, there is an urgent need to construct highly accurate bioinformatics tools for gender inference from TGS data and reporting sex chromosomal abnormalities.

**TABLE 1:** Gender prediction accuracy of different methods for samples in dataset 1.

Tools	Accuracy for all samples (%)	Accuracy for male samples (%)	Accuracy for female samples (%)
PLINK	81.44	48.28	100
seXY	62.5	45.45	81.63
XYalign	98.08	100	95.92
seGMM	99.52	100	98.98

In this study, we verified the performance and accuracy of the new gender inference tool, seGMM, using both in-house and publicly available TGS, WES, and WGS datasets. The seGMM tool used unsupervised learning to integrate the information of the X and Y chromosomes from the TGS, WES, or WGS datasets and classified the samples into one of the six sex chromosomal karyotypes (XX, XY, XYY, XXY, XXX, and X).

## Materials and Methods

### Data

We compared the performances of three existing gender-inferring methods and seGMM using the TGS data from the following 3 datasets: 1) Dataset 1: exon-targeted sequencing data of 1,000 genes (34 X chromosomal genes and two Y chromosomal genes) for a cohort of 110 males and 98 females from the 1,000 Genomes Project (Supplementary Table S1) (Genomes Project et al., 2010); 2) Dataset 2 (in-house): massive parallel sequencing of 785 deafness-related genes (eight genes in the X chromosome) for an in-house cohort of 8,805 males and 7,582 females; and 3) Dataset 3: targeted sequencing data of 187 autism risk genes (13 genes in the X chromosome) for a cohort of 42 females and 205 males from the Autism Clinical and Genetic Resources in China (ACGC) (Guo et al., 2018).

We also used the following two publicly available datasets (Supplementary Tables S2, S3) and one in-house dataset for analyzing the performance of seGMM in determining gender using WES and WGS data: 1) Dataset 4: exome sequencing data of 164 males and 118 females from the 1,000 Genomes Project (Genomes Project et al., 2015); 2) Dataset 5 (in-house): exome sequencing data of 1,257 males and 1,136 females; and 3) Dataset 6: high-coverage whole genome sequencing data of 11 males and 16 females from the 1,000 Genomes Project (Genomes Project et al., 2015).

The publicly available BAM files were previously mapped to the reference genome (GRCh37) and directly used for downstream analyses. For the in-house datasets, Fastp was used to remove the adapters and low-quality reads, and the quality of sequencing data was evaluated using measures such as Q20, sequence duplication levels, coverage, and GC content (Chen et al., 2018). Clean DNA sequencing reads were mapped to the human reference genome (GRCh37) using the BWA-MEM algorithm (Li and Durbin, 2009). Duplicated reads in the BAM files from the public and in-house datasets were removed using the sambamba tool (Tarasov et al., 2015). The variants were identified based on the Genome Analysis Toolkit best practices recommendations (McKenna et al., 2010) and filtered with VCFtools (Danecek et al., 2011) using parameters such as missing data in more than 50% of samples, minor allele count <3, overall SNP quality (QUAL) score <30, and read depth <5.

### Gender Inference Using seGMM

The model for seGMM included five gender-associated features, namely, X chromosome heterozygosity (XH), reads mapped to the X chromosome (Xmap), reads mapped to the Y chromosome (Ymap), the ratio of X/Y counts (XYratio), and the mean depth of the sex-determining region of the Y chromosome (*SRY*) gene (SRY_dep). The seGMM tool computed XH as the fraction of all genotypes on the X chromosome with two different allele calls, excluding the missing genotypes. Xmap/Ymap was computed as the fraction of high-quality reads (mapq > 30) that mapped to the X/Y chromosome divided by the total number of high-quality reads that mapped to the genome using the samtools algorithm (Li et al., 2009). XYratio was computed as the ratio of Xmap to Ymap (Xmap/Ymap). SRY_dep was determined using the mosdepth tool (Pedersen and Quinlan, 2018). The seGMM tool allows the users to customize feature selection for the GMM model because different TGS panel designs may only provide some features. For example, if the gene panel contains only genes located on the X chromosome, the relevant features on the X chromosome (XH and Xmap) are extracted and put into the model for gender determination.

**FIGURE 1:**
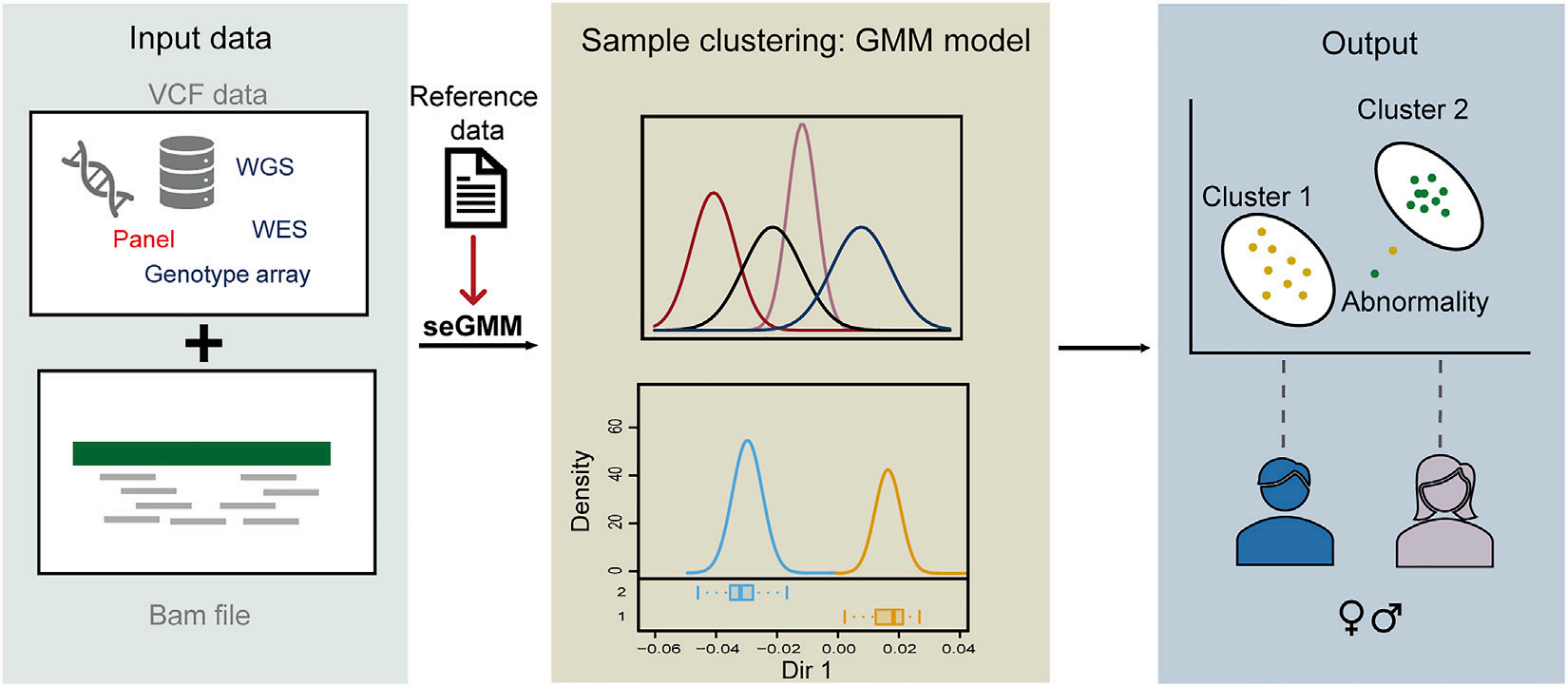
Schematic diagram of seGMM. The seGMM tool automatically collects features from the input VCF and BAM files and builds the GMM model. The output of seGMM includes gender prediction results and identification of samples with abnormal sex chromosomes.

The features extracted from the BAM and VCF files were normalized to the same level using the scale function in R 4.1.2 (R Core Team., 2021). Then, the mclust (v.5.4.9) R package was used to perform model-based clustering with the expectation-maximization (EM) algorithm and the samples were classified into two clusters (Scrucca et al., 2016). The gender was inferred based on the cluster results for a group of samples. The outliers were identified when uncertainty (probability of being assigned to two different clusters) was greater than 0.1. When a single sample was submitted, gender was inferred using the reference data that was analyzed with the same features as those in the seGMM model (Figure 1).

### Identifying Potential Sex Chromosomal Abnormalities in the Sequenced Samples

We defined the gates to classify individual karyotypes. The distribution of Xmap and Ymap in the females and males of the large cohort was normal. The ratio of samples with sex chromosomal abnormalities was 0.022% (Nielsen and Wohlert, 1990). This data was in agreement with the empirical rule, which states that 99.7% of normally distributed data lies within 3 standard deviations (sd) of the mean. Hence, we defined the normal gates as mean±3sd. The fold changes in Xmap or Ymap values indicated sex chromosomal aneuploidy.

To identify sex chromosomal abnormalities in the samples, we first calculated the mean value and standard deviation values of Xmap (mean_xmap and sd_xmap) and Ymap (mean_ymap, and sd_ymap) in the genetically determined male and female samples. The values for the males and females were denoted as m and f, respectively. The following six gates were then used to classify the karyotypes of individuals:

• XY Gate
  ○ mean_xmap_m - 3 sd_xmap_m < x < mean_xmap_m + 3 sd_xmap_m
  ○ mean_ymap_m - 3 sd_ymap_m < y < mean_ymap_m + 3 sd_ymap_m
• XYY gate:
  ○ mean_xmap_m - 3 sd_xmap_m < x < mean_xmap_m + 3 sd_xmap_m
  ○ y > 2 mean_ymap_m
• XX gate:
  ○ mean_xmap_f - 3 sd_xmap_f < x < mean_xmap_f + 3 sd_xmap_f
  ○ mean_ymap_f - 3 sd_ymap_f < y < mean_ymap_f + 3 sd_ymap_f
• XXY gate:
  ○ x > 2 mean_xmap_f
  ○ mean_ymap_m - 3 sd_ymap_m < y < mean_ymap_m + 3 sd_ymap_m
• XXX gate:
  ○ x > 3 mean_xmap_f
  ○ mean ymap_f - 3 sd_ymap_f < y < mean_ymap_f + 3 sd_ymap_f
• X gate:
  ○ x < 0.5 mean_xmap_f
  ○ mean_ymap_f - 3 sd_ymap_f < y < mean_ymap_f + 3 sd_ymap_f

### Comparing the Performance of seGMM With Other Existing Gender-Inference Methods

The performance of seGMM was compared to PLINK 1.9, XYalign (v.1.1.6), and seXY (v.20170316). For PLINK 1.9, the pseudoautosomal region of the X chromosome was first split off with the parameter--split-x. Then, the parameter--check-sex was run without parameters. After reviewing the distribution of F estimates, the parameter--check-sex was rerun with parameters corresponding to the empirical gap. XYalign was performed following the method described in the original literature. The CHROM_STATS module was used to obtain the depths of chromosomes 1, X, and Y. The depths of X and Y chromosomes were normalized relative to the depth of chromosome 1. Then, a scatter plot of normalized X and Y chromosomes depth was plotted to assess gender in samples. Gender of the samples was inferred with seXY using the X.ped and Y.ped data that was derived from PLINK. The training dataset was provided by seXY. We expected to compare seGMM and other existing tools for all the six datasets. However, target gene panel data for datasets 2 and 3 did not contain genes on the Y chromosome. Therefore, the performance of XYalign and seXY was not available for these two datasets.

### STR Analysis for Verifying Gender

The STR analysis was performed using the customized multiplex PowerPlex® 16 System, which allowed co-amplification and four-color detection of amelogenin and other gene loci. The following primers were used for amplifying amelogenin: forward, 5′- GTT​AG​ACG​TGT​GCT​TCA​ACT​TCA​GCT​ATG​AGG​TAA​TTT​TTC—3′; reverse, 5′- ATC​CGA​CGG​TAG​TGT​CCA​ACC​ATC​AGA​GCT​TAA​ACT​GG-3′. All genetic loci were amplified simultaneously in a single tube and analyzed in a single lane. One of the primers for the amelogenin gene was labeled with carboxyrhodamine (ROX). The amplicons were separated in the ABI 3730XL Genetic Analyzer and the data was extracted using GeneMapper ID v3.2. The gender was inferred according to the peaks for the amelogenin gene. If only one peak was observed for the amelogenin locus, the gender was designated as female. If two distinct peaks differing by 6 bp were observed in the amelogenin locus, the gender was designated as male.

### Quantitative Determination of Y Chromosome Copy Number

Genomic DNA (gDNA) was extracted using the MagMAX High Purity Free DNA Separation Kit (Magen, China). DNA concentration of the samples was measured using the NanoDrop One spectrophotometer (Thermo Fisher Scientific, United States). The working concentration of all DNA samples was 20 ng/µl. The primers targeting *SRY*, zinc finger protein Y-linked (*ZFY*), and deleted in azoospermia 1 (*DAZ1*) genes were designed using the Primer-BLAST online tool to determine the Y chromosome copy number (Ye et al., 2012). *RPP30* was used as the internal control. All the primers used in this study are listed in Supplementary Table S4. The qPCR reaction mix included 0.6 µl of gDNA, 0.4 µl of each primer, 5 µl of iTaq™ Universal SYBR® Green Supermix (Bio–Rad, United States), and 3.6 µl of double-distilled water. Each sample was analyzed with three replicates. The quantitative real-time PCR assay (RT–qPCR) was performed in the QuantStudio 5 Real-Time PCR system (Thermo Fisher Scientific) using the following conditions: initial hot start cycle at 98°C for 2 min followed by 40 cycles consisting of denaturation at 98°C for 10 s, annealing at 60°C for 10 s, and the final extension step of 30 s at 72°C.

## Results

### The seGMM Tool Shows Better Performance Compared to Other Tools for the TGS Data

**FIGURE 2:**
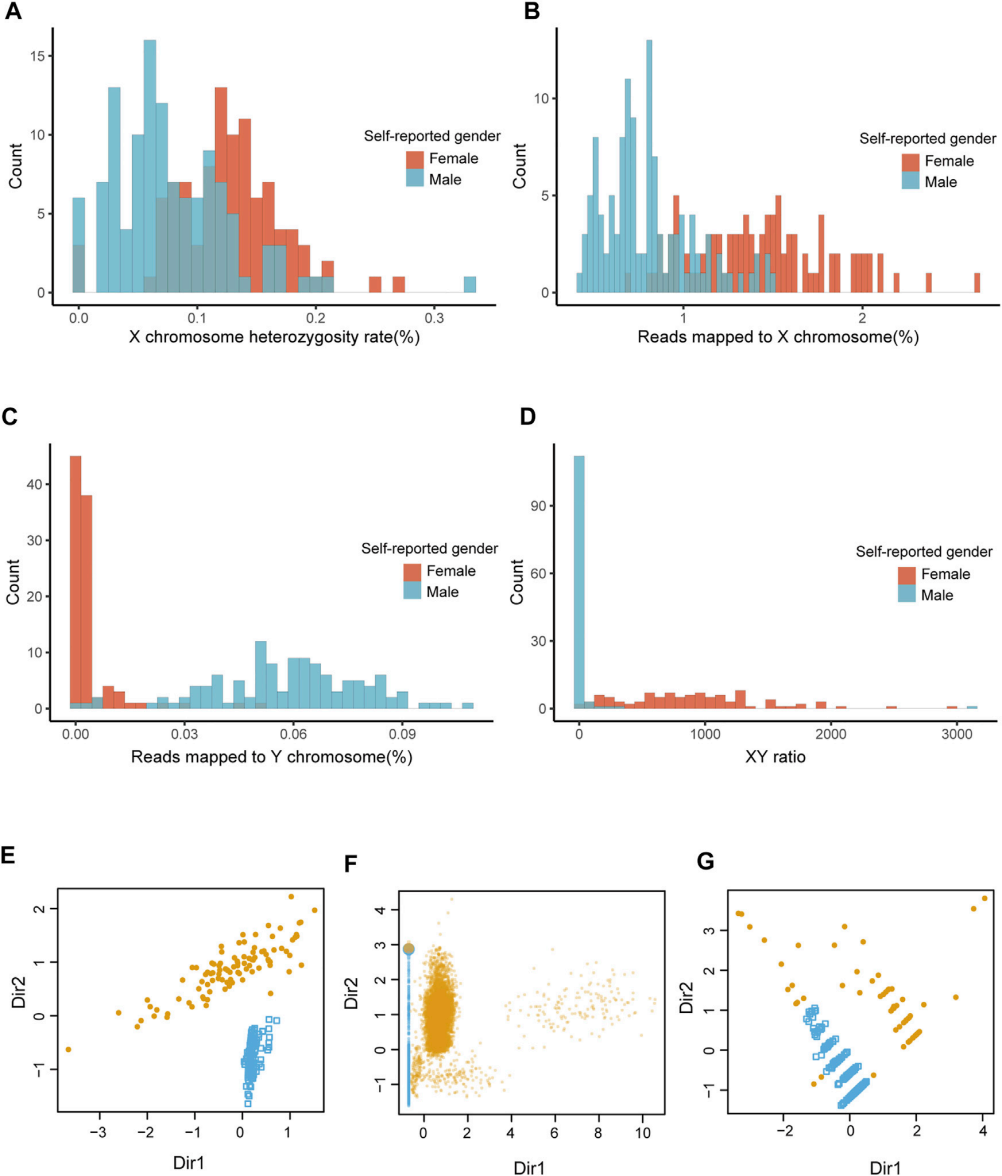
The performance of seGMM in the TGS datasets. **(A–D)** Distribution of features collected from dataset 1. **(E–G)** Sample classification results of datasets 1, 2, and 3 based on seGMM. The colors represent different sample clusters. Dir1 and Dir2 represent the eigenvectors that specify the discriminant subspace generated from the features included in the GMM model.

The distribution of XH, Xmap, Ymap, and XYratio values for dataset 1 (*n* = 208) shown in Figures 2A–D. The accuracy of seGMM was 99.52% and none of the samples were outliers (Figure 2E; Table 1). The accuracy of seGMM in females and males was 98.98 and 100%, respectively. The XYratio of one female sample (NA19054) resembled that of males and was incorrectly classified as male by seGMM. The gender-inference performance of seGMM for dataset 1 was superior to PLINK, seXY and XYalign. The PLINK tool analysis showed that the F coefficients for the dataset1 samples ranged from 0 to 0.9 and gap of F coefficients was not observed (Supplementary Figure S1). The accuracy of PLINK was 81.44% by running --check-sex without parameters. The accuracy of seXY for the dataset 1 was only 62.5% (Table 1). XYalign does not directly indicate predicted gender. Therefore, plotting the normalized sequence depth of the sex chromosomes and cluster samples along two ellipses using the stat_ellipse function resulted in a confidence level of 99.99%. XYalign plot showed that one female sample was located along with the male samples and three female samples were located between the two ellipses. Hence, the predicted gender of these four female samples was ambiguous (Supplementary Figure S2) and the accuracy of XYalign is 98.08%.

**FIGURE 3:**
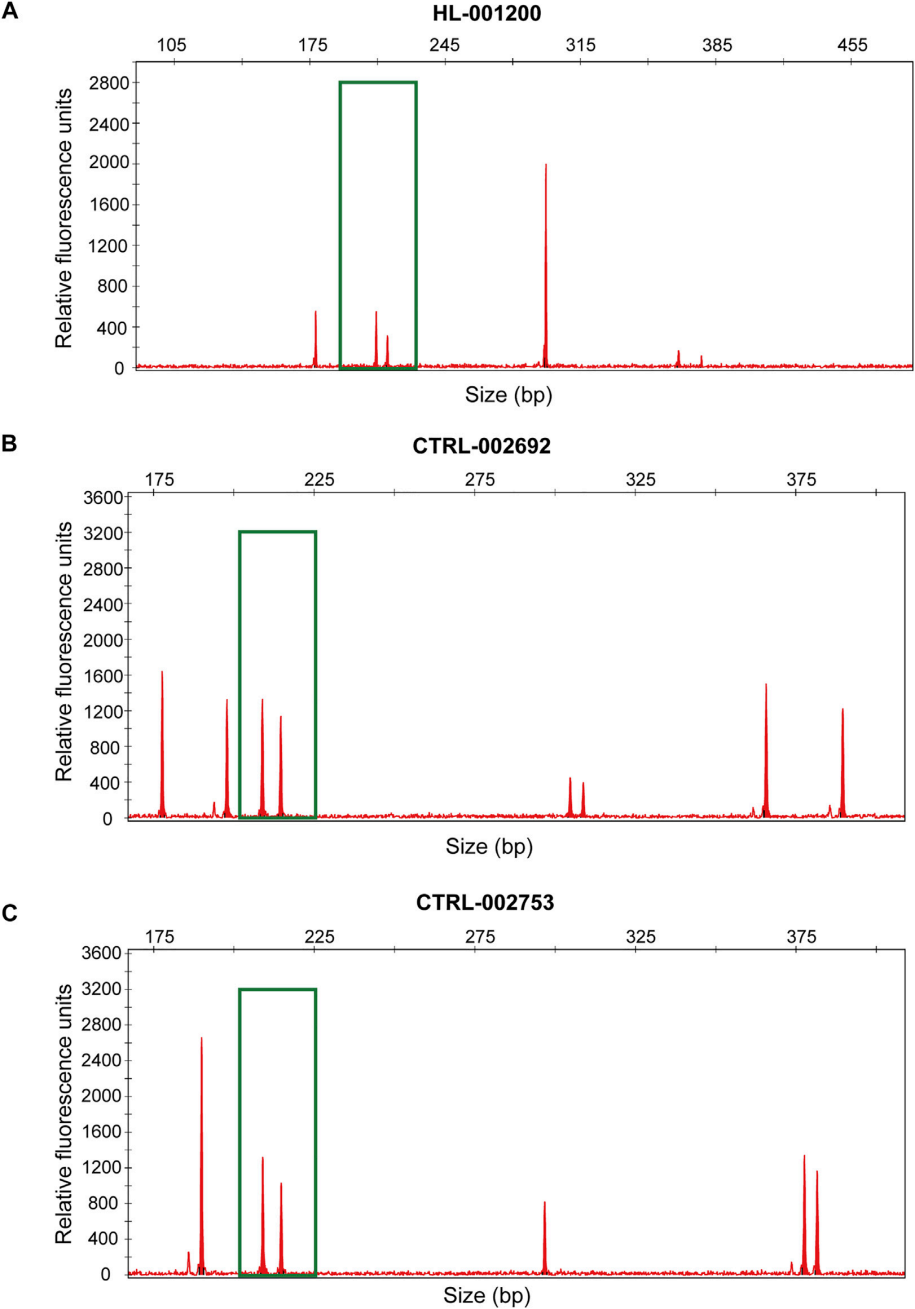
Experimentally verified gender of HL-001200 **(A)**, CTRL-002692 **(B)** and CTRL-002753 **(C)**. The green box shows the location of the amelogenin loci.

The performance of seGMM for the target gene panel data was validated using dataset 2 (*n* = 16,387) and dataset 3 (*n* = 247). The read counts, base quality, and GC distribution of the sequencing data of all the 16,387 subjects in dataset 2 was assessed. The average total number of sequence read per sample was 10.72 million. The average quality score for all bases was above 30 and the average GC content was 50.11% per subject. The average targeted sequence coverage was 90.43%, and unique mapping rate of each sample was 99.26%. We identified 16,988 variants in eight genes located on the X chromosome. Because the target gene panel for dataset 2 did not contain genes on the Y chromosome, we only used XH and Xmap to analyze the performance of the seGMM model. XH and Xmap plots showed distinct clusters for males and females (Supplementary Figure S3). The overall accuracy of seGMM and PLINK was 99.92 and 87.10%, respectively (Figure 2F; Table 2). The accuracy of seGMM in females and males was both 99.98%. One self-reported female sample (HL-001200) and two self-reported male samples (CTRL-002692 and CTRL-002753) were misclassified. Therefore, we performed STR analysis with the sex chromosome marker gene, amelogenin, to verify the gender of these three ambiguous samples. All these three samples were identified as males because two distinct peaks were observed with a difference of 6 bp for the amelogenin gene (Figures 3A–C). CTRL-002692 and CTRL-002753 were misclassified because all other male samples had a XH value of 0, while these two samples had a non-zero value (XH_CTRL-002692_ = 0.0025; XH_CTRL-002753_ = 0.0046), which could be caused by the individual variation in targeting region.

**TABLE 2:** Gender prediction accuracy of different methods for samples in datasets 2 and 3.

Methods	Dataset 2	Dataset 3
PLINK	87.10	38.87
seGMM	99.92	92.31

The overall accuracy of seGMM for dataset 3 was 92.31% (97.56% for females and 84.88% for males, Figure 2G and Table 2). The accuracy of PLINK was only 38.87% for dataset 3. The performances of seGMM and PLINK were significantly better for datasets 1 and 2 compared to dataset 3 because the number of X chromosome SNPs (81) were lower and sequencing data for the Y chromosome was absent in dataset 3, thereby affecting the distribution of XH values from the male and female samples (Supplementary Figure S4A). In contrast to PLINK, seGMM collected additional information for the reads mapped to the X chromosome, thereby enabling better separation between the female and male samples (Supplementary Figure S4B). Furthermore, we assessed the performance of seGMM using features only extracted from the X chromosome in dataset 1. The seGMM tool showed that 59 samples were outliers and the accuracy for the remaining samples was only 84.56%. We then evaluated the computation time of different methods using 1 core, 10 cores and 20 cores on a server with 64 Intel(R) Xeon(R) CPU E7-8895 v3 at 2.60 GHz. The analysis time for the seGMM tool was longer than PLINK and seXY because it collected additional features such as reads mapped to the X and Y chromosomes. Moreover, the analysis time for seGMM with 1 core was longer than XYalign and 10 times faster than XYalign with 20 cores (Supplementary Table S5).

### The seGMM Tool Shows Better Accuracy Than Other Known Tools for the WES and WGS Data

We then evaluated the performance of the seGMM tool for the WES and WGS data. First, we analyzed the publicly available WES data (dataset 4). The accuracy of seGMM was 100% for the samples in dataset 4 (*n* = 282) (Table 3 and Supplementary Figure S5). The accuracy of PLINK and seXY was also 100%. The accuracy of XYalign was 99.65% (Supplementary Figure S6).

**TABLE 3:** Gender prediction accuracy of different methods for the WES and WGS datasets.

Datasets	PLINK	XYalign	seXY	seGMM
1000G phase3 WES data	100	99.65	100	100
1000G phase3 high quality WGS data	100	100	100	100
In-house WES data	99.79	99.91	49.23	100

**FIGURE 4:**
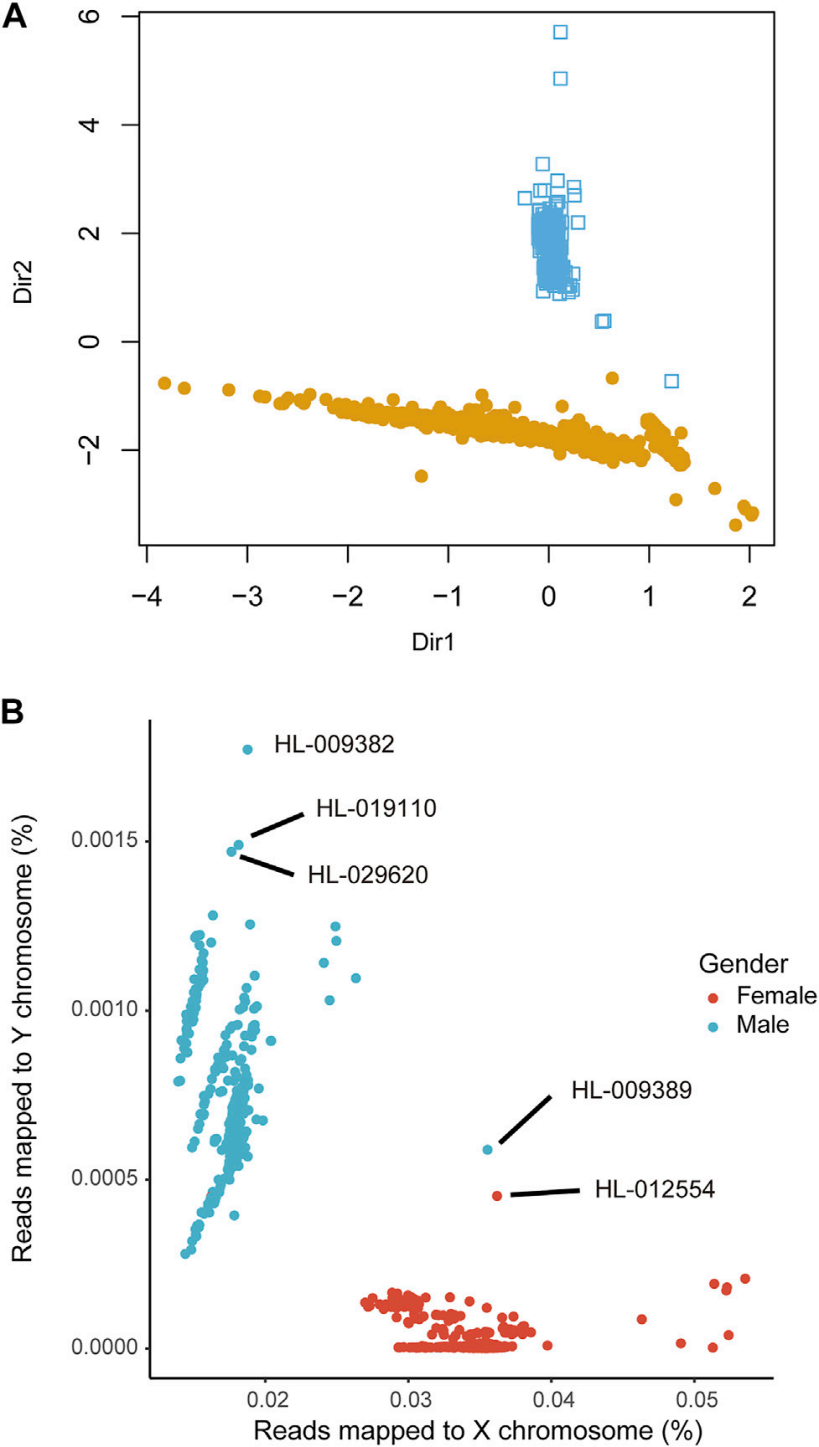
The prediction accuracy of seGMM in inferring the gender of samples from the in-house WES dataset. **(A)** Sample clustering results of seGMM. The colors represent different sample clusters. Dir1 and Dir2 represent eigenvectors that specify the discriminant subspace generated from the features included in the GMM model. **(B)** Scatter plot shows the reads mapped to the X and Y chromosomes. As shown, we identified three samples (HL-029620, HL-009382 and HL-019110) with XYY sex chromosome karyotypes.

Next, we analyzed the in-house WES data (dataset 5, *n* = 2,393) using seGMM and other tools. In dataset 5, the average number of sequencing reads per sample was 114.43 million. The average Q20, Q30 and GC content of the reads per subject was 97.36%, 93.21%, and 51.23%, respectively. Furthermore, the average unique mapping rate for each individual sample was 99.92%. We identified 89,273 variants on the X chromosome and 4,866 variants on the Y chromosome. The concordance between inferred gender and self-reported gender using the seGMM tool based on the five features for the in-house WES dataset 5 was 99.75% (99.76% for males and 99.74% for females, Figure 4A, Supplementary Figure S7). Six mismatched samples (HL-005584, HL-006009, HL-006904, HL-007335, HL-007935 and HL-012246) were identified by comparing SNP-inferred gender and self-reported gender. This indicated misregistration of clinical information for some samples. Therefore, we performed STR analysis to validate the gender of these six samples. Three samples were classified as females because they showed only one peak for the amelogenin locus, whereas the remaining three samples showed two distinct peaks with a difference of 6 bp and were classified as males (Table 4). The results demonstrated that the actual accuracy of seGMM prediction was 100%. We also evaluated the correlation between age and reads mapped to the Y chromosome in the male samples (Supplementary Figure S8A) and reads mapped to the X chromosome in the female samples from the in-house WES dataset 5 (Supplementary Figure S8B). The results showed significant negative correlation (*p* = 6.899e-09; correlation coefficient: −0.17) between reads mapped to the Y chromosome and age, thereby indicating loss of Y chromosome during aging.

**TABLE 4:** Experimental verification of gender prediction results for samples in the in-house WES data.

Sample ID	Size of PCR products in the amelogenin loci (bp)	Self-reported gender	seGMM inferred gender	Experimentally validated gender
HL-005584	209.15	Male	Female	Female
	-			
HL-006009	209.06	Male	Female	Female
	-			
HL-006904	209.04	Female	Male	Male
	214.8			
HL-007335	209.06	Female	Male	Male
	214.85			
HL-007935	209.11	Male	Female	Female
	-			
HL-012246	209.18	Female	Male	Male
	214.92			
HL-033182	209.02	Female	Female	Female
	-			
HL-020292	209.19	Female	Female	Female
	-			
HL-011500	209.25	Male	Male	Male
	215.07			
HL-019211	209.19	Male	Male	Male
	215.04			
HL-009389	209.27	Female	Female	Female
	-			
HL-012554	209.26	Male	Male	Male
	215.03			

We then compared the performances of PLINK, seXY and XYalign for dataset 5 using the corrected gender information. The accuracy of PLINK was 99.79% with five mismatched samples (HL-033182, HL-020292, HL-011500, HL-019211 and HL-012554) (Table 3). The accuracy of XYalign was 99.91% with two mismatched samples (HL-009389 and HL-012554). Overall, six samples were mismatched, as predicted by PLINK and XYalign. STR analysis showed that the gender of these samples was consistent with their self-reported gender and matched the predicted results of seGMM analysis (Table 4). Furthermore, the accuracy of seXY was 49.23%. The loss of accuracy in seXY for dataset 5 was because the distribution of Y chromosome missingness in the male and female samples were confounded (Supplementary Figure S9). Finally, the performances of these tools were assessed using the WGS data (dataset 6, *n* = 27). The accuracy of all tools was 100% for dataset 6 (Table 3).

### The seGMM Tool Identifies Samples With Sex Chromosomal Abnormalities

The seGMM tool can identify six sex chromosomal karyotypes (XX, XY, XYY, XXY, XXX, and X) using Xmap and Ymap. In a large cohort, the distribution of Xmap and Ymap was normal in females and males. The Xmap or Ymap values of samples with sex chromosome abnormalities such as XYY and XXY were significantly different and were recognized as outliers compared to samples with normal sex chromosomes. Three samples in dataset 5 (HL-029620, HL-009382 and HL-019110) were classified as the XYY karyotype. In dataset 5, the average rate of reads mapping to the X and Y chromosomes in the female samples were 0.035 ± 0.0020 and 1.61e-05 ± 3.36e-05, respectively, and 0.018 ± 0.0011 and 0.00067 ± 0.00014, respectively, for the male samples. The rate of reads mapping to the Y chromosome for the three outlier samples was twice as high as the mean value of Ymap in all the male samples (Ymap_HL-029620_ = 0.0015, Ymap_HL-009382_ = 0.0018, and Ymap_HL-019110_ = 0.0015), thereby suggesting a XYY karyotype by seGMM (Figure 4B). Furthermore, although HL-009389 and HL-012554 samples were located close together in the middle of the plot, they were correctly predicted by seGMM as female and male, respectively (Figure 4B). This is because features such as Xmap and SRY_dep, which are not shown in Figure 4B, clearly separated all female and male samples (Supplementary Figure S10). This demonstrated the significance of incorporating key features to improve the accuracy of the gender prediction model. In the other datasets, sex chromosomal abnormalities were not identified.

**FIGURE 5:**
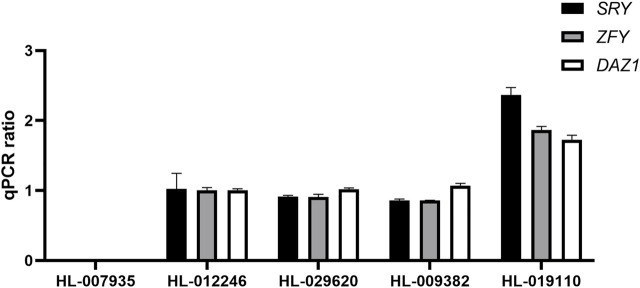
Quantitative determination of Y chromosome copy number.

Next, we evaluated the accuracy of the data-based sex chromosome karyotype of these three samples by analyzing the copy number ratios of Y chromosome-specific genes (*SRY*, *ZFY* and *DAZ1*) by RT–qPCR. We used HL-007935 and HL-012246 samples as controls for females and males based on the STR analysis results. The copy number ratio for normal females was 0. The copy number ratio for normal male samples was 1. We analyzed the copy number ratios of HL-029620, HL-009382 and HL-019110 samples in dataset 5 and found that the copy number ratio of HL-019110 was 2 (Figure 5). This confirmed that the karyotype for the HL-019110 sample was XYY.

## Discussion

In this study, we characterized the performance of the new gender inference tool, seGMM, in comparison with the other established gender inference tools using NGS data, especially TGS panel data. The seGMM tool used unsupervised clustering to classify samples based on X and Y sex chromosomal features. The performance and accuracy of the seGMM tool were significantly better than other existing gender inference tools using TGS, WES, and WGS data. Furthermore, seGMM accurately predicted six different sex chromosomal karyotypes, including those with sex chromosome abnormalities. The mean and standard deviation values of Xmap and Ymap were used to determine potential sex chromosome aneuploidy in the male and female samples by seGMM. Previous studies have identified sex chromosomal aneuploidy in samples by measuring the intensities of X and Y chromosomes (Bycroft et al., 2018; Turro et al., 2020). A similar strategy was incorporated into the seGMM tool and used to validate a sample with sex chromosome karyotype XYY in the in-house WES dataset. Samples with sex chromosomal abnormalities may result in false calling of the genotype. This can affect identification of pathogenic variants in the sex chromosomes. Therefore, samples with sex chromosome abnormalities should be removed or recalled genotypes to ensure accuracy of the clinical diagnosis.

The seGMM tool applies unsupervised learning algorithm to infer gender of samples from the TGS panel data to overcome the pitfalls of existing tools. The TGS panel consists of a select set of genes with known or suspected association with the disease under study. The advantage of TGS in clinical genetic testing includes high sequencing depth of the genes of interest, which allows identification of rare and causative variants (Eggers et al., 2016; Bewicke-Copley et al., 2019). The data size of TGS depends on the number of genes included in the panel and the methods used for targeted sequencing including target enrichment by hybridization capture and amplicon sequencing. Hence, the number of variants and sequencing depth of the X and Y chromosomes varies for different TGS panels. The accuracy of existing methods in inferring gender using TGS data is unsatisfactory because the algorithms are either based on a data-dependent threshold or supervised learning on a fixed sample set (Purcell et al., 2007; Qian et al., 2017). PLINK uses a data-dependent threshold strategy that determines gender by computing the F coefficients based on the observed and expected number of homozygous markers and requires reasonable minor allele frequency estimates. However, variants detected in the TGS datasets tend to have lower minor allele frequency and the number of variants detected in the X chromosome are limited. Therefore, F coefficient of the male and female samples based on the TGS data is ambiguous. Furthermore, the logistic regression classifier for the seXY tool was based on GWAS data collected from prostate cancer and ovarian cancer samples, and was not suitable for TGS panels because the distribution of X chromosome heterozygosity and Y chromosome missingness varied between the TGS panel dataset and the training dataset. In contrast, seGMM applied a Gaussian mixture model to infer gender. Therefore, the performance and accuracy of seGMM were higher for data with different covariance structures and were adaptable to include fresh samples.

Our study also demonstrated that the gender-inference accuracy of seGMM improved when the information from both X and Y chromosomes was available. For example, the accuracy of seGMM for dataset 1 was 84.56% when the data included only X chromosomal features, but the accuracy increased to 99.52% upon adding Y chromosomal features. Moreover, the accuracy of seGMM was lower for male samples compared to female samples in datasets 2 and 3 because the sequencing data did not contain information on genes in the Y chromosome. Our data also suggested that addition of probes that target unique regions of the Y chromosome such as the *SRY* exon, which is involved in typical male sex development (Gubbay et al., 1990; Parma and Radi, 2012), is helpful for inferring genders using the TGS panel data.

DNA sequencing data from the lymphoblastoid cell lines (LCLs) established from the EBV-infected peripheral blood mononuclear cells (PBMCs) may confound the prediction of sex chromosomal karyotypes. A previous study demonstrated that EBV transformation adversely affected the genomic DNA stability; mosaic loss of X chromosome was observed in 7% (2/29) of the samples analyzed (Shirley et al., 2012). The false-positive rates due to EBV-induced mutations in LCLs may reduce the accuracy of predicting the sex chromosomal karyotypes. The majority of samples in the 1000G WES data were derived from LCLs, but we did not identify any sample in this dataset with abnormal sex chromosomal aneuploidy. The box plots of reads mapped to the Y chromosome showed a much lower value for one male sample (NA12413) compared to the others, thereby indicating potential loss of chromosome Y (Supplementary Figure S11). However, we could not confirm if the loss of Y chromosome was due to LCLs or as a result of authentic sex chromosome abnormalities since experimental validation is required for further analysis.

A few critical considerations are necessary while applying seGMM. First, seGMM is not applicable when the targeted sequencing data does not include genes located on the X and Y chromosomes. Secondly, seGMM requires a sufficient sample size to train an accurate model. Therefore, prediction accuracy should be enhanced for small sample datasets by including reference data (using --reference function parameter). We have provided two reference datasets that were generated from the 1000G WES and WGS datasets. In addition, samples sequenced with the same version of TGS panel can be used to build a user’s own reference to maximize the accuracy of gender prediction. When applying seGMM, the experimental and analytical methods between reference data and testing data need to be consistent to prevent bias. Thirdly, parallel computing (using --num_threshold function parameter) is recommended to speed up the analysis since the seGMM tool collects more features than the other existing tools. Lastly, the use of Empirical Rule to classify individual karyotypes improves the recall rate, but may magnify false positives rate, as has been reported in previous study using this strategy (Turro et al., 2020). In addition, many factors may contribute to false positive predicition results, including the copy number variations such as large deletions or insertions on the sex chromosomes or genetic chimerism. Therefore, to overcome this limitation, karyotyping of predicted abnormal samples is recommended to confirm the sample karyotype.

In conclusion, we demonstrate that the performance and accuracy of seGMM, a new tool to infer sex chromosomal karyotypes based on a Gaussian mixture model, was significantly higher and satisfactory for TGS, WES, and WGS datasets, including those with samples containing sex chromosomal abnormalities compared to other existing tools. Hence, seGMM is a promising tool for inferring the gender of samples in TGS, WES, and WGS datasets.

## Data Availability

The original contributions presented in the study are included in the article/Supplementary Material, further inquiries can be directed to the corresponding authors.
